# *In vitro* and *in vivo* evaluation of diethyldithiocarbamate with copper ions and its liposomal formulation for the treatment of *Staphylococcus aureus* and *Staphylococcus epidermidis* biofilms

**DOI:** 10.1016/j.bioflm.2023.100130

**Published:** 2023-05-17

**Authors:** Laurine Kaul, Adrian I. Abdo, Tom Coenye, Simon Swift, Andrew Zannettino, Regine Süss, Katharina Richter

**Affiliations:** a Richter Lab, Department of Surgery, Basil Hetzel Institute for Translational Health Research, University of Adelaide, 37 Woodville Road, Adelaide, SA, 5011, Australia; b Institute of Pharmaceutical Sciences, Department of Pharmaceutics, University of Freiburg, Sonnenstr. 5, 79104, Freiburg, Germany; c Adelaide Medical School, Faculty of Health and Medical Sciences, University of Adelaide, 4 North Terrace, Adelaide, SA, 5000, Australia; d Laboratory of Pharmaceutical Microbiology, Ghent University, Ottergemsesteenweg 460, 9000, Ghent, Belgium; e Department of Molecular Medicine and Pathology, University of Auckland, 85 Park Road, Grafton, Auckland, 1023, New Zealand; f Precision Cancer Medicine Theme, South Australian Health & Medical Research Institute, North Terrace, Adelaide, SA, Australia; g Central Adelaide Local Health Network, Adelaide, Australia; h Institute for Photonics and Advanced Sensing, University of Adelaide, Adelaide, Australia

**Keywords:** Biofilms, Surgical site infections, Diethyldithiocarbamate, Copper ions, Liposomes, *Staphylococcus aureus*, *Staphylococcus epidermidis*

## Abstract

Surgical site infections (SSIs) are mainly caused by *Staphylococcus aureus* (*S. aureus*) and *Staphylococcus epidermidis* (*S. epidermidis*) biofilms. Biofilms are aggregates of bacteria embedded in a self-produced matrix that offers protection against antibiotics and promotes the spread of antibiotic-resistance in bacteria. Consequently, antibiotic treatment frequently fails, resulting in the need for alternative therapies. The present study describes the *in vitro* efficacy of the Cu(DDC)_2_ complex (2:1 M ratio of diethyldithiocarbamate (DDC^−^) and Cu^2+^) with additional Cu^2+^ against *S. aureus* and *S. epidermidis* biofilms in models mimicking SSIs and *in vitro* antibacterial activity of a liposomal Cu(DDC)_2_ + Cu^2+^ formulation. The *in vitro* activity on *S. aureus* and *S. epidermidis* biofilms grown on two hernia mesh materials and in a wound model was determined by colony forming unit (CFU) counting. Cu^2+^-liposomes and Cu(DDC)_2_-liposomes were prepared, and their antibacterial activity was assessed *in vitro* using the alamarBlue assay and CFU counting and *in vivo* using a *Galleria mellonella* infection model. The combination of 35 μM DDC^−^ and 128 μM Cu^2+^ inhibited *S. aureus* and *S. epidermidis* biofilms on meshes and in a wound infection model. Cu(DDC)_2_-liposomes + free Cu^2+^ displayed similar antibiofilm activity to free Cu(DDC)_2_ + Cu^2+^, and significantly increased the survival of *S. epidermidis*-infected larvae. Whilst Cu(DDC)_2_ + Cu^2+^ showed substantial antibiofilm activity *in vitro* against clinically relevant biofilms, its application in mammalian *in vivo* models is limited by solubility. The liposomal Cu(DDC)_2_ + Cu^2+^ formulation showed antibiofilm activity *in vitro* and antibacterial activity and low toxicity in *G. mellonella*, making it a suitable water-soluble formulation for future application on infected wounds in animal trials.

## Graphical abstract

**Figure:**
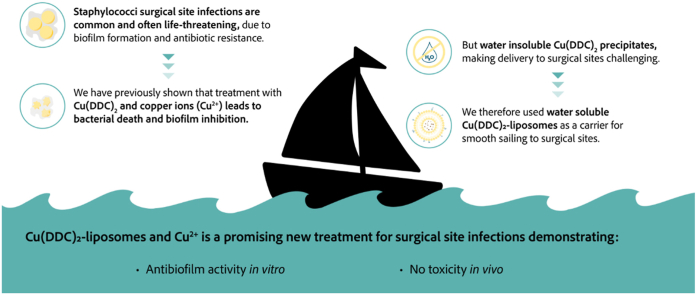

## 1 Introduction

Surgical site infections (SSI) are amongst the most common surgery-associated infections and occur in 1.5–20% of surgeries, depending on the nature of the surgery and country in which it is performed [1]. SSIs develop at the organ/tissue site of surgery [2] and can range from wound or implant infections to organ infections [3]. Following a surgical procedure, such as hernia mesh repair [4], infections can affect the incision site (from superficial to deep tissue), implanted material and any part of the anatomy that was exposed or manipulated during surgery [[5], [6], [7]]. Consequently, SSIs represent a significant burden, by increasing patient morbidity and mortality, and adding additional cost to health systems [2,3,5].

The most common pathogens associated with SSIs are *Staphylococcus aureus* (*S. aureus*) and coagulase negative staphylococci, including *Staphylococcus epidermidis* (*S. epidermidis*), which are natural components of the respiratory tract and skin microbiota, respectively [8]. Therefore, prevention of SSIs requires pre-operative preparations of the surgical site and antibiotic prophylaxis [2]. If an infection is detected, the routine treatment relies on additional antibiotic therapy [9,10]. However, over the last two decades, the antibiotic missuse and overuse has promoted the emergence of resistant strains, such as methicillin resistant *S. aureus* (MRSA). The situation is exacerbated by biofilm infections, which are frequently staphylococcal, that offer antibiotic tolerance [11,12]. Biofilms are aggregates of bacteria embedded in a protective matrix, which enables bacteria to persist in hostile conditions, communicate with each other and become highly tolerant to antibiotics [13]. In comparison to planktonic forms of bacteria, biofilm bacteria require 10 to 1000-fold higher concentrations of antibiotics to be eradicated [14]. This is a major concern, as biofilms are present in over 80% of SSIs and are a major cause of delayed wound healing [10]. In addition, patient mortality is increased by 2 to 11-fold in MRSA-associated SSIs, compared to susceptible *S. aureus* associated SSIs and surgeries without infections [15]. Therefore, there is an unmet need for new antimicrobial agents targeting MRSA and *S. epidermidis* biofilms to prevent and treat SSIs.

Diethyldithiocarbamate (DDC^−^) is a metabolite of disulfiram, a drug used for the treatment of chronic alcoholism [16], that is being repurposed for the treatment of cancer (Clinicaltrials.gov Identifier: NCT04234022, NCT05210374) and infections caused by parasites [[17], [18], [19]], viruses [20], fungi [[21], [22], [23]] and bacteria [[24], [25], [26], [27]]. The anticancer and antibacterial activity of DDC^−^ is associated with the formation of complexes with metal ions, with copper ions (Cu^2+^) being the most effective [25,[28], [29], [30]]. The combination of DDC^−^ and Cu^2+^ was antibacterial against *Mycobacterium tuberculosis* [25], *Streptococcus pneumoniae* [30] and was previously extended to planktonic *S. aureus* and *S. epidermidis* and their biofilms [31]. At a concentration of 35 μM DDC^−^ and 128 μM Cu^2+^, the combination inhibited multiple steps in the biofilm formation cycle, reduced *S. aureus* and *S. epidermidis* biofilm viability and showed high fibroblast cell viability *in vitro*. These concentrations correspond to the instant formation of the Cu(DDC)_2_ complex [2 mol DDC^−^:1 mol Cu^2+^] and additional Cu^2+^, and displayed *in vivo* efficacy and non-toxicity in an invertebrate model [31].

However, the antibacterial activity of 35 μM DDC^−^ and 128 μM Cu^2+^ was only observed on biofilms grown in a microtiter well plate over 24 h [31] and can alter when exposed to biofilms grown over multiple days or in conditions similar to SSIs [32]. In addition, the Cu(DDC)_2_ complex is insoluble (<0.1 mg/ml) in water, limiting its practicality in the clinical setting [33]. This necessitates the development of a pharmaceutical formulation for optimal drug delivery to infection sites and improved antibacterial efficacy. To improve the solubility of Cu(DDC)_2_, nanoparticles including liposomal formulations of Cu(DDC)_2_ have been developed and successfully used as therapeutically active agents against cancer cells [[33], [34], [35], [36], [37]], with enhanced activity against breast cancer cells [38], glioblastoma [39] and neuroblastoma cells [40].

Inspired by this, our aim was to evaluate the antibacterial properties of 35 μM DDC^−^ and 128 μM Cu^2+^ (Cu(DDC)_2_ + Cu^2+^) in biofilm models mimicking SSIs and to develop an appropriate drug delivery vehicle for Cu(DDC)_2_ to enable clinical application of the combination. Thus, this study advances our previous knowledge by presenting, for the first time, the antibiofilm activity of Cu(DDC)_2_ + Cu^2+^ against *S. aureus* and *S. epidermidis* in an *in vitro* implant and wound infection model. Furthermore, we have validated the non-toxicity and efficacy of the liposomal Cu(DDC)_2_ + Cu^2+^ formulation *in vivo* using a *Galleria mellonella* infection model.

## 2 Methods and materials

### 2.1 Bacterial strains, mesh materials and chemicals

*S. aureus* ATCC 6538, *S. aureus* ATCC 700699 (also known as MRSA Mu50) and *S. epidermidis* ATCC 35984 were purchased from the American Type Culture Collection (Manassas, VA, USA). Bacteria were inoculated at colony forming unit (CFU)/ml or optical density at 600 nm (OD_600_) values stated after dilution of an overnight culture grown in tryptone soya broth (TSB) or nutrient broth (Thermo Fisher Scientific, Waltham, MA, USA) at 37 °C with shaking at 180 rpm. Tryptone soya agar (TSA) was prepared by adding 1.5% agar bacteriological (Thermo Fisher Scientific). The hernia meshes Parietex Hydrophilic 2-Dimensional mesh (polyester), Parietene Lightweight monofilament polypropylene mesh (polypropylene) were donated by Covidien (Dublin, Ireland). The saturated phospholipids 1,2-distearoyl-*sn*-glycero-3-phosphocholine (DSPC) and 1,2-distearoyl-*sn*-glycero-3-phosphoethanolamine-*N*-[methoxy (polyethylene glycerol)-2000] (DSPE-mPEG2000) were donated by Lipoid GmbH (Ludwigshafen, Germany). Unless stated otherwise, all chemicals, materials, media and supplements were purchased from Sigma-Aldrich (Steinheim, Germany).

### 2.2 Biofilm formation on hernia meshes

Round coupons (1.5 cm diameter) of polyester and polypropylene meshes were placed in a 12-well plate and immersed in 2 ml of a bacterial suspension (2 × 10^6^ CFU/ml) of *S. aureus* ATCC 6538, MRSA Mu50 or *S. epidermidis* ATCC 35984 in TSB and incubated at 37 °C on a rotating platform at 70 rpm (3D Gyratory Mixer; Ratek Instruments, Boronia, Australia). After 24 h incubation, meshes with attached bacteria were washed by immersing the meshes into 3 ml 0.9% (w/v) saline for 30 s at room temperature, three times consecutively, and placed into fresh TSB. Following another 72 h incubation, the meshes were washed, as previously described with 0.9% saline, to remove loosely attached cells and placed into TSB solutions containing 35 μM DDC^−^ + 128 μM Cu^2+^. Control wells contained TSB alone (untreated control). Following 24 h treatment incubation at 37 °C on a rotating platform (70 rpm), a third washing step was performed prior to CFU counting or imaging of the coupons.

For CFU counting, meshes were collected in 10 ml 0.9% saline and biofilms were extracted from the mesh and disrupted by a series of vortexing (5 min, maximum speed, VM1 Vortex Mixer, Ratek Instruments Pty Ltd, Victoria, Australia) and sonication (15 min, Soniclean 80TD, Pulse swept power 60 W, Soniclean Pty Ltd, South Australia, Australia), prior to serial dilution and plating on TSA. CFU were counted following 24 h incubation at 37 °C. For imaging, the last washing step was performed with phosphate buffered saline. Meshes were covered and incubated with a 1:500 dilution of LIVE/DEAD BacLight staining (1:1 mix of SYTO 9/propidium iodide; Life Technologies, Scoresby, Australia) in TSB for 20 min in the dark and imaged using the Olympus FV1000 Live cell imaging system (Olympus, Shinjuku, Japan) and a 20 × /0.5 W objective. Quantitation of live/dead cells was performed using ImageJ software (NIH, Bethesda, MA, USA). Briefly, the contrast/brightness was adjusted globally to images to minimize background before setting a threshold to highlight cells for automated counting.

### 2.3 *In vitro* wound model

An artificial dermis made of collagen (Corning, NY, USA) and hyaluronic acid (1.20–1.80 MDa; Lifecore Biomedical, MN, USA) was prepared as previously described by Brackman et al. [41]. According to established protocols [42], freeze-dried bovine plasma was rehydrated in 10 ml 0.9% saline, 19 ml Bolton broth (LabM, Lancashire, UK), 1 ml freeze-thaw laked horse blood (Biotrading, Mijdrecht, Netherlands) and 20 μl heparin 100 IU. An artificial dermis was placed in each well of a 24-well plate and soaked with 1 ml of this mixture. Then, an overnight culture of MRSA Mu50 or *S. epidermidis* ATCC 35984 in TSB adjusted to an OD_600_ 0.1, was diluted 1:100 in 0.9% saline, 10 μl were added on top of each dermis (equal to 10^4^ CFU/well) and incubated statically at 37 °C for 24 h. Following biofilm formation, 1 ml of 35 μM DDC^−^ + 128 μM Cu^2+^ in TSB was added. Controls included biofilms exposed to TSB (untreated control). After 24 h treatment exposure, each dermis was placed in 10 ml of 0.9% saline, and biofilms were extracted from the dermis and disrupted by three consecutive vortexing and sonication cycles for 30 s each. After serial dilution, plating on TSA and incubation at 37 °C for 24 h, CFU were counted to determine antibiofilm activity.

### 2.4 Liposomal preparation

Cu^2+^-liposomes and Cu(DDC)_2_-liposomes composed of DSPC:Cholesterol:DSPE-mPEG_2000_ [50:45:5 M ratio] were produced and characterized according to Hartwig et al. [40]. Briefly, lipid films were prepared with the thin film hydration method and hydrated with an aqueous Cu^2+^ solution (150 mM) to obtain a lipid concentration of 40 mM. Subsequently, the Cu^2+^-lipid mix was extruded for 41 passages through an 80 nm pore-sized polycarbonate membrane (GE Healthcare Life Science, Marlborough, MA, USA) at 65 °C. Separation of non-encapsulated Cu^2+^ from Cu^2+^-liposomes was achieved by size exclusion chromatography with a Sephadex G-50 Fine (GE Healthcare Life Science) column equilibrated with an EDTA containing sucrose buffer (300 mM sucrose, 20 mM HEPES, 30 mM EDTA, pH 7.4). Buffer exchange to an EDTA-free sucrose buffer (300 mM sucrose, 20 mM HEPES, pH 7.4) was performed through three centrifugation steps (3000×*g*, room temperature, 1.5 h) using Vivaspin® Turbo 4 filtration units (100 kDa MWCO; Sartorius AG, Göttingen, Germany), followed by Cu^2+^-liposomes collection.

Cu(DDC)_2_-liposomes were prepared by complexation of DDC^−^ with the liposomal encapsulated Cu^2+^ at 25 °C/300 rpm (Thermomixer comfort, Eppendorf, Hamburg, Germany) for 10 min. Excess of DDC^−^ was removed by three centrifugation steps (3000×*g*, room temperature, 45 min) with EDTA-free sucrose buffer. Non-incorporated Cu(DDC)_2_ precipitated and was separated from the Cu(DDC)_2_-liposomes by prefiltration through a 0.45 μm cellulose acetate filter (VWR International, Radnor, PA, USA) before and after the centrifugation steps.

Cu^2+^-liposomes and Cu(DDC)_2_-liposomes were stored at 4–6 °C for up to 3 months and were sterile filtered under aseptic conditions through a 0.2 μm cellulose acetate filter (VWR International) before use. As previously described by Hartwig et al. [40], the hydrodynamic diameter (d_h_) and the polydispersity index (PDI) were measured via dynamic light scattering (ZetaPals, Brookhaven Instruments Corporation, Holtsville, NY, USA) and encapsulated Cu^2+^ concentrations were determined by measuring absorbance of complexed Cu^2+^ with DDC^−^ in methanol at a wavelength of *λ*_max_ = 435 nm with a GENESYS 10S UV–Vis spectrophotometer (Thermo Fisher Scientific). Liposomes were used in biofilm challenge experiments to provide the equivalent of 35 μM DDC^−^ and/or 128 μM Cu^2+^.

### 2.5 Antibacterial activity of liposomes

Overnight cultures of MRSA Mu50 and *S. epidermidis* 35984 in nutrient broth were adjusted to an OD_600_ 0.5 and further 1:15 (v/v) diluted in nutrient broth. Black-walled 96-well microtiter plates (Greiner Bio-one, Frickenhausen, Germany) were inoculated with 100 μl bacterial suspension and incubated for 24 h at 37 °C on a rotating platform at 70 rpm. The biofilm was rinsed with 0.9% saline, exposed to 100 μl of Cu(DDC)_2_-liposomes, Cu^2+^-liposomes, [Cu(DDC)_2_-liposomes + Cu^2+^-liposomes], [Cu(DDC)_2_-liposomes + free Cu^2+^] or 35 μM DDC^−^ + 128 μM Cu^2+^ and further incubated for 24 h under the same conditions. The treatments were removed, and the biofilm rinsed with 0.9% saline, before viability was detected by either measurement of metabolic activity with the alamarBlue assay or CFU counting.

The alamarBlue assay was performed according to Richter et al. [43] and rinsed biofilms were incubated with a 10% (v/v) alamarBlue™ Cell Viability Reagent (Thermo Fisher Scientific) solution in nutrient broth. The fluorescence was measured hourly on a TECAN Spark plate reader (Männedorf, Switzerland) at *λ*_*excitation*_ = 530 nm/*λ*_*emission*_ = 590 nm until maximum fluorescence was reached, then viability was calculated using Equation (1). Antibiofilm activity of the different treatments was determined as percentage of biofilm viability, where the fluorescence intensity of treated and untreated biofilms is represented by I_treated_ and I_untreated_, respectively and I_blank_ represents the background fluorescence of the 10% v/v alamarBlue solution [43].

(1) $$\%\;Biofilm\;viability=\left(\frac{I_{treated}-I_{blank}}{I_{untreated}-I_{blank}}\right)\times 100$$

CFU counting was performed according to Van den Driessche et al. [44] and 100 μl of 0.9% saline were added to each rinsed biofilm. To disrupt the biofilm, the plates were shaken at 150 rpm and sonicated (5 min each), and the content of each well was collected separately. This process was repeated twice to extract all biofilms cells and serial dilutions of these suspensions were plated on TSA and incubated at 37 °C for 24 h, prior to CFU counting.

### 2.6 *In vivo* cytotoxicity and antibacterial activity

*Galleria mellonella* (*G. mellonella*) larvae (Angel-Zentrum, Freiburg, Germany) were used on the day of receipt and 30 larvae were assigned to each treatment group. Larvae were injected in the last left proleg with micro-fine (30 gauge) needle insulin syringes (BD, Franklin Lakes, NJ, USA). Four control groups were included, (i) not-injected larvae (uninfected, untreated control), (ii) larvae injected with 0.9% saline (uninfected, vehicle control), (iii) larvae injected with treatment (uninfected, treated control to determine toxicity) and (iv) larvae injected with a bacterial suspension and 0.9% saline (infected, vehicle control). To determine treatment efficacy, larvae were injected with a *S. epidermidis* ATCC 35984 suspension (OD_600_ 0.05) in nutrient broth and with Cu(DDC)_2_-liposomes, Cu^2+^-liposomes, [Cu(DDC)_2_-liposomes + Cu^2+^-liposomes] or [Cu(DDC)_2_-liposomes + free Cu^2+^]. Considering the dilution factor within the larvae, the concentrations of the liposomal formulations were increased 10-fold compared to the concentrations used *in vitro*. A total volume of 20 μl was injected comprising treatment or 0.9% saline in a 1:1 mix with a bacterial suspension in nutrient broth. Larvae were housed in petri dishes in the dark at 37 °C and the larvae survival was monitored daily over 4 days.

### 2.7 Statistical analysis

Results were statistically analyzed using GraphPad Prism version 9.00 for Windows (GraphPad Software, CA, USA) and statistical significance was determined with an α = 0.05. All experiments were carried out at least in triplicate. Parametric data are represented by the mean ± standard deviation (SD), which was analyzed using paired 2-tailed t-tests, one-way analysis of variance (ANOVA) with Dunnett's multiple comparison test for finding differences between treatment groups and untreated controls and two-way ANOVA with Šidák's multiple comparison tests, as described in the figure legends. *G. mellonella* survival data was analyzed using Kaplan-Meier survival curves with significant differences between groups determined by log-rank test, significance was Bonferroni-Holm-corrected for multiple comparisons.

## 3 Results

### 3.1 Treatment of biofilms on hernia mesh materials

When we consider the antibacterial properties of Cu(DDC)_2_ + Cu^2+^ observed in microtiter plates possibly not correlating with complex biofilms present in SSIs [30], we used two biofilm models mimicking SSIs to further investigate the antibiofilm activity of 35 μM DDC^−^ + 128 μM Cu^2+^ *in vitro*. These models are based on an implant infection and a wound infection.

**Fig. 1:**
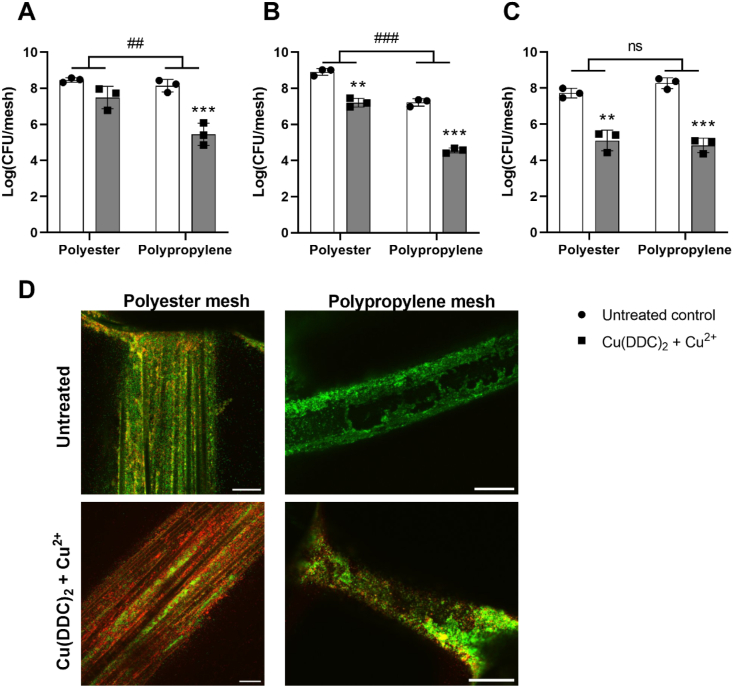
Effect of 35 μM diethyldithiocarbamate (DDC^−^) + 128 μM Cu^2+^ (grey; Cu(DDC)_2_ + Cu^2+^) on biofilms grown on hernia mesh material. Log_10_ colony forming units (CFU) of (A) *S. aureus* ATCC 6538, (B) MRSA Mu50 and (C) *S. epidermidis* ATCC 35984 biofilms grown on Parietex Hydrophilic 2-Dimensional (polyester) or on Parietene Lightweight monofilament polypropylene (polypropylene) meshes compared to untreated control (white; n = 3; mean ± SD; 2-way ANOVA: **p < 0.01, ***p < 0.001 indicate significant differences between Cu(DDC)_2_ + Cu^2+^ and untreated control by Šidák's multiple comparison test; ##p < 0.01, ###p < 0.001 indicate significant differences between the polyester and the polypropylene mesh; ns = not significant). (D) To visually illustrate the quantitative culture-based cell-viability data, the effect of Cu(DDC)_2_ + Cu^2+^ on *S. aureus* ATCC 6538 biofilms were investigated using confocal microscopy of LIVE/DEAD BacLight stained meshes. Confocal microscopy images result: green = viable bacteria; red = dead bacteria. Z-stack images taken with a 20 × /0.5 W objective are representative of three independent experiments. Scalebar indicated on bottom-right of images correspond to 75 μm. (For interpretation of the references to color in this figure legend, the reader is referred to the Web version of this article.)

As an example of SSI on an implant, we investigated the biofilm formation of *S. aureus* and *S. epidermidis* on two commonly used, commercially available, hernia mesh materials and the ability of Cu(DDC)_2_ + Cu^2+^ to reduce the bacterial load on these meshes. *S. aureus* ATCC 6538, MRSA Mu50 and *S. epidermidis* ATCC 35984 formed extensive biofilms during 96 h batch incubations on polyester and polypropylene mesh material with log (CFU/mesh) values ranging from 7.21 to 8.91 (Fig. 1). The imaging of *S. aureus* ATCC 6538 biofilms on polyester meshes showed a multifilament mesh structure, exhibiting niches for bacteria to attach (Fig. 1D, top left). In contrast, the mono filaments of the polypropylene mesh were surrounded by *S. aureus* ATCC 6538 biofilms (Fig. 1D, top right). Studies suggest that staphylococci biofilms on hernia meshes may be associated with hernia repair failure and contribute to mesh shrinkage, chronic pain or hernia recurrence [45], and there may be an association between mesh porosity and the formation of biofilms [46].

When treated with 35 μM DDC^−^ + 128 μM Cu^2+^, viability of *S. aureus* ATCC 6538 in biofilms was reduced on polyester and polypropylene meshes (Fig. 1A). Similar results were observed in MRSA Mu50 (Fig. 1B) and *S. epidermidis* ATCC 35984 (Fig. 1C) biofilms log_10_ reduction on polyester meshes and polypropylene meshes. The *S. aureus* ATCC 6538 and MRSA Mu50 log_10_ reduction was higher on polypropylene meshes compared to polyester meshes. This could be due to multifilament meshes forming denser biofilms than monofilament meshes because of the increased surface and presence of niches [47]. In addition, the highly hydrophobic Cu(DDC)_2_ complex that is formed instantly when DDC^−^ and Cu^2+^ are mixed, might not reach the bacteria embedded in the niches of the multifilament mesh.

The imaging of Cu(DDC)_2_ + Cu^2+^ treated *S. aureus* ATCC 6538 (Fig. 1D, bottom left) confirmed a substantial number of bacteria in the niches formed by the intertwined filaments but showed mostly dead bacteria (red) on the polyester mesh and was associated with CFU reduction. In contrast, the *S. aureus* ATCC 6538 biofilm that previously surrounded the polypropylene filaments was in parts removed during washing steps, resulting in only few dead bacteria (red) imaged (Fig. 1D bottom right). We quantified the viability based on the percentage of green and red fluorescent cells, which showed the viability was reduced when treated with Cu(DDC)_2_ + Cu^2+^ compared to the untreated control on polyester and polypropylene meshes (Supplementary file 1). However, significant background was present due to autofluorescence of the polyester and polypropylene that compose the meshes, which significantly affected automated counting of live and dead cells. This was unavoidable since further background removal would eliminate valid signal from the analysis. Therefore, the microscopy images visually complement the quantitative assessment of log_10_ reduction of bacteria due to Cu(DDC)_2_ + Cu^2+^. As the overall successful salvage rate of infected meshes can be as low as 10% and be inferior for infected polyester mesh compared to polypropylene mesh [4], the substantial log_10_ reduction of Cu(DDC)_2_ + Cu^2+^ on both mesh material highlights the combination as a promising treatment approach for infected hernia meshes.

### 3.2 Efficacy in an *in vitro* wound model

**Fig. 2:**
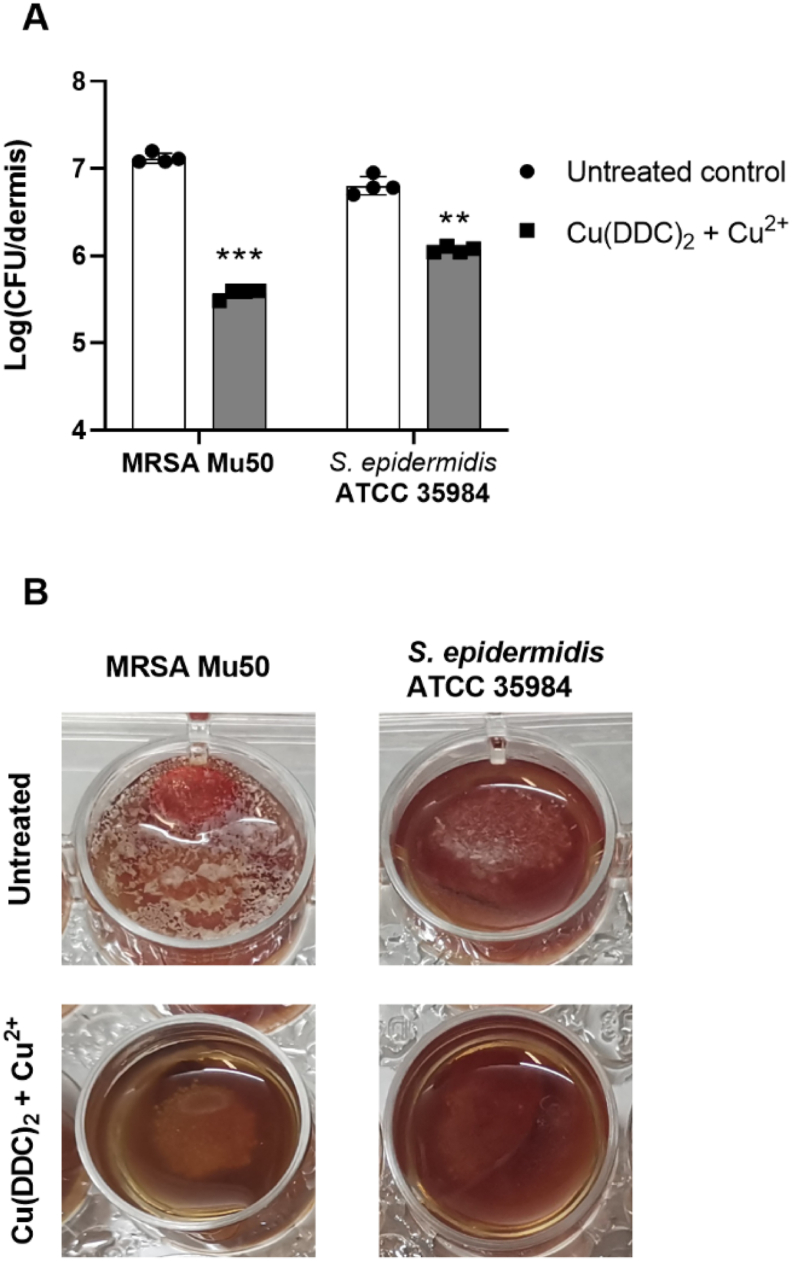
Effect of 35 μM diethyldithiocarbamate (DDC^−^) + 128 μM Cu^2+^ on MRSA Mu50 and *S. epidermidis* ATCC 35984 biofilms grown on an artificial dermis compared to the untreated control. (A) Log (CFU/dermis) of untreated biofilms (white) and biofilms treated with Cu(DDC)_2_ + Cu^2+^ (grey; n = 4; mean ± SD; paired 2-tailed t-tests: **p < 0.01, ***p < 0.001). (B) Representative images of MRSA Mu50 (left) and *S. epidermidis* ATCC 35984 (right) biofilms when untreated (top) or treated with Cu(DDC)_2_ + Cu^2+^ (bottom).

As second *in vitro* SSI model, the artificial dermis model was chosen, as it closely resembles a chronic wound infection with similar nutritional conditions found in wound exudate and a dermis-like scaffold based on hyaluronic acid and collagen on which bacteria can attach and form biofilms [41,48]. Here, MRSA Mu50 and *S. epidermidis* ATCC 35984 biofilms were grown on an artificial dermis and exposed to 35 μM DDC^−^ + 128 μM Cu^2+^ (Fig. 2). The combination of Cu(DDC)_2_ + Cu^2+^ demonstrated a significant biofilm reduction in MRSA Mu50 and in *S. epidermidis* ATCC 35984 biofilms (Fig. 2A). While the log_10_ reduction was smaller compared to the mesh attachment model for both MRSA and *S. epidermidis* biofilms, Cu(DDC)_2_ + Cu^2+^ exposure still visually reduced the biofilms on the artificial dermis (Fig. 2B) and resulted in 97.2% and 81.5% MRSA Mu50 and *S. epidermidis* ATCC 35984 reduction, respectively, despite nutrient rich *in vivo*-like conditions. We propose three explanations for a reduced exposure of Cu(DDC)_2_ + Cu^2+^ with the biofilm on the artificial dermis.

Firstly, when DDC^−^ and Cu^2+^ solutions are mixed, the water insoluble Cu(DDC)_2_ complex precipitates and sediments to the bottom of the well [49]. In previous biofilm experiments, including the biofilm on mesh material, biofilms were grown or placed at the bottom of wells, allowing for precipitated Cu(DDC)_2_ to sediment onto and interact with the biofilms, while excess Cu^2+^ was available in solution. In the wound model, biofilms are formed on top of the artificial dermis at the air-liquid interface (Fig. 2B). Therefore, when exposed to Cu(DDC)_2_ + Cu^2+^, limited amount of Cu(DDC)_2_ would precipitate onto the biofilm on the artificial dermis, while the remaining Cu(DDC)_2_ might interact with the hydrophobic collagen or simply sediment to the bottom of the well. Secondly, Cu^2+^ was shown to increase cross-linking of collagen in a concentration dependent matter [50], which can result in a reduced availability of Cu^2+^ for the antibiofilm activity. Lastly, DDC^−^ can be degraded to diethylamine and carbon sulfide in the presence of blood, due to the presence of plasma proteins and may therefore not be available to form the Cu(DDC)_2_ complex [51]. Similar effects of the microenvironmental conditions in the artificial dermis model on the antibiofilm activity of antimicrobial agents were reported [42,48,52]. For example, Grassi et al. [48] observed inferior biofilm inhibition by antimicrobial peptides in the artificial dermis model compared to a 3D lung epithelial model due to the presence of blood and proposed the development of nanocarriers as drug delivery vehicle [53]. Consequently, to increase water solubility of Cu(DDC)_2_, prevent Cu(DDC)_2_ sedimentation and protect DDC^−^ from degradation, Cu^2+^ and Cu(DDC)_2_ were incorporated into PEGylated liposomes.

### 3.3 Characterization of Cu^2+^-liposomes and Cu(DDC)_2_-liposomes

**Fig. 3:**
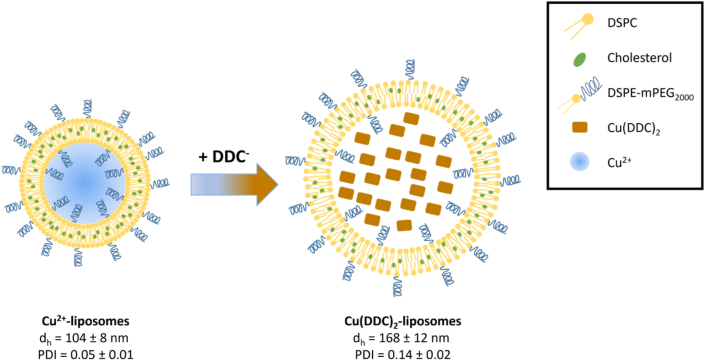
Schematic illustration of Cu^2+^-liposomes and Cu(DDC)_2_-liposomes. Diethyldithiocarbamate (DDC^−^) diffuses through the membrane of the smaller Cu^2+^-liposomes and binds the encapsulated Cu^2+^ to form the water insoluble Cu(DDC)_2_. The trapped Cu(DDC)_2_ accumulates within the liposome, resulting in an increase in size. DSPC = 1,2-distearoyl-*sn*-glycero-3-phosphocholine; DSPE-mPEG_2000_ = 1,2-distearoyl-*sn*-glycero-3-phosphoethanolamine-*N*-[methoxy (polyethylene glycerol)-2000]; d_h_ = hydrodynamic diameter; PDI = polydispersity index (n = 15; mean ± SD).

PEGylated Cu^2+^-liposomes and Cu(DDC)_2_-liposomes were prepared and characterized according to Hartwig et al. [40]. The size, expressed as the d_h_, and the PDI were determined for Cu^2+^-liposomes and Cu(DDC)_2_-liposomes (Fig. 3) and were similar to previously reported values [40]. The size of both the Cu^2+^-liposomes and the Cu(DDC)_2_-liposomes were below 200 nm, allowing for sterile filtration and excluding the presence of large aggregates and extra-liposomal Cu(DDC)_2_ [40]. In addition, the PDI of Cu^2+^-liposomes and Cu(DDC)_2_-liposomes was below 0.2, indicating a homogenous population of liposomes [54,55], which has previously been confirmed by imaging of mostly unilamellar vesicles in cryo-electron microscopy images [33,40]. The production of Cu(DDC)_2_-liposomes is based on DDC^−^ diffusing through the membrane of Cu^2+^-liposomes and forming the insoluble Cu(DDC)_2_ complex within the liposomes, which is characterized by the color change [49]. In addition, Wehbe et al. [33] showed that the amount of Cu(DDC)_2_ in liposomes correlates with the amount of Cu^2+^ in liposomes by comparing Cu^2+^ to lipid ratio to Cu(DDC)_2_ to lipid ratio. Therefore, it can be assumed that both liposomes have the same lipid constitution and consequently a similar amount of PEG polymers per liposome. Based on this assumption, the different sizes of the liposomes and the homogenous vesicle population, the PEGylation of Cu^2+^-liposomes would be denser compared to Cu(DDC)_2_-liposomes (Fig. 3).

### 3.4 Antibiofilm activity of liposomal Cu(DDC)_2_ + Cu^2+^

**Fig. 4:**
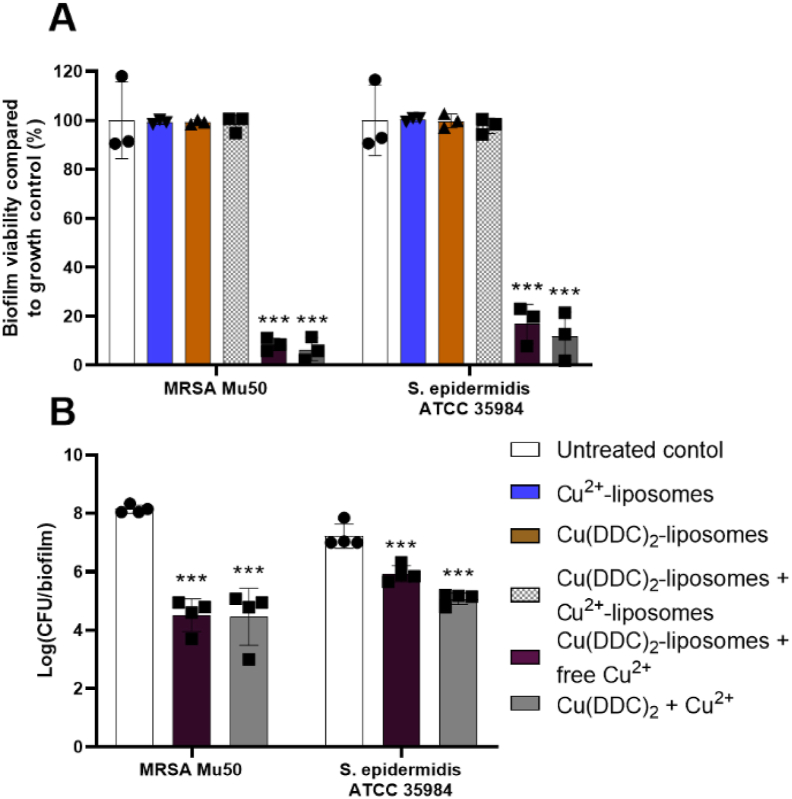
Effect of Cu^2+^*-*liposomes, Cu(DDC)_2_-liposomes, [Cu(DDC)_2_-liposomes + Cu^2+^-liposomes], [Cu(DDC)_2_-liposomes + free Cu^2+^] and Cu(DDC)_2_ + Cu^2+^ (35 μM DDC^−^ + 128 μM Cu^2+^) on MRSA Mu50 and *S. epidermidis* ATCC 35984 biofilm viability in comparison to the untreated control by using (A) the alamarBlue assay and (B) colony forming unit (CFU) counting. The concentrations of Cu(DDC)_2_-liposomes and Cu^2+^-liposomes or the combinations correspond to 35 μM diethyldithiocarbamate (DDC^−^) and/or 128 μM Cu^2+^, respectively (n = 3–4; mean ± SD; 1-way ANOVA: ***p < 0.001 by Dunnett's multiple comparison tests).

The liposomes were assessed for their activity against MRSA Mu50 and *S. epidermidis* ATCC 35984 biofilms (Fig. 4). As a fast and high throughput method [44], the alamarBlue assay was first performed to determine antibiofilm activity of the liposomal formulations (Fig. 4A). Treatment with Cu^2+^-liposomes or Cu(DDC)_2_-liposomes showed no activity against MRSA Mu50 and *S. epidermidis* ATCC 35984 biofilms. Similar to the effects of free Cu^2+^ and Cu(DDC)_2_ on MRSA and *S. epidermidis* biofilms [31], Cu^2+^-liposomes and Cu(DDC)_2_-liposomes concentrations up to a 4-fold increase did not inhibit biofilm viability (data not shown). The combination of [Cu(DDC)_2_-liposomes + Cu^2+^-liposomes] also showed no antibiofilm activity against MRSA Mu50 and *S. epidermidis* ATCC 35984. This could be a result of the Cu(DDC)_2_-liposomes, the Cu^2+^-liposomes or both liposomes not releasing their content extracellularly or, following bacterial uptake, intracellularly. However, cellular uptake of PEGylated Cu(DDC)_2_-liposomes were observed in LS cells after 6 h incubation [40], which suggest bacterial uptake of the Cu(DDC)_2_-liposomes. Notably, when Cu(DDC)_2_-liposomes were investigated in combination with free Cu^2+^ [Cu(DDC)_2_-liposomes + free Cu^2+^], the biofilm viability of MRSA Mu50 and *S. epidermidis* ATCC 35984 was significantly reduced. This reduction in biofilm viability was similar to the activity of free Cu(DDC)_2_ + Cu^2+^ against MRSA Mu50 and *S. epidermidis* ATCC 35984 biofilms. To further confirm these results, CFU counting was performed for treatments showing a reduction in biofilm viability with the alamarBlue assay (Fig. 4B). Treatment with [Cu(DDC)_2_-liposomes + free Cu^2+^] and Cu(DDC)_2_ + Cu^2+^ resulted in a significant MRSA Mu50 log_10_ reduction and a significant *S. epidermidis* ATCC 35984 log_10_ reduction. As the antibiofilm activity of [Cu(DDC)_2_-liposomes + free Cu^2+^] against MRSA and *S. epidermidis* was similar to free Cu(DDC)_2_ + Cu^2+^ and treatment with free Cu^2+^ alone previously showed no antibiofilm activity against MRSA Mu50 and *S. epidermidis* ATCC 35984 at the tested concentration [31], we concluded that Cu(DDC)_2_ was released from the Cu(DDC)_2_-liposomes, either intracellularly following bacterial uptake or extracellularly, but not the uncomplexed Cu^2+^ from the Cu^2+^-liposomes.

Liposomes can penetrate the biofilm and release their content by fusing with the bacterial phospholipid membrane [56,57]. This interaction is dependent on biofilm properties, including bacterial species and matrix composition, and by the liposomal physicochemical properties [56]. Liposomes vary in surface charge, lipid composition, bilayer rigidity, surface modification, size and the incorporation of PEG polymers in the liposomal membrane [58,59]. As Cu(DDC)_2_-liposomes are produced by DDC^−^ diffusion into Cu^2+^-liposomes, it can be expected that Cu(DDC)_2_-liposomes and Cu^2+^-liposomes have the same lipid constitution [33] and are only different in size and membrane PEGylation density. The denser PEGylation of the Cu^2+^-liposomes compared to the Cu(DDC)_2_-liposomes (Fig. 3) can present a physical barrier for Cu^2+^-liposome interaction with bacterial membranes or biofilm matrix, and therefore, prevent the intracellular uptake of the liposomal content [58]. PEGylated liposomes were previously shown to reduce interaction with target cells [60] and limit interactions with bacterial biofilms [61]. Liposomes with a PEGylated surface showed improved penetration of *Pseudomonas aeruginosa* biofilms but reduced the affinity of liposomes to bacteria compared to non-PEGylated liposomes. The PEG modifications on the liposome surface increase hydrophilicity of liposomes which increased the affinity to biofilm matrix components, such as extracellular polymeric substance [59]. In addition, PEGylated DSPC-containing liposomes with a low surface charge and rigid bilayer reduce adsorption of the DSPC-liposomes on *S. aureus* biofilms compared to non-PEGylated liposomes [61]. To investigate if the PEG polymers are hindering adsorption of Cu^2+^-liposomes on MRSA and *S. epidermidis* biofilms and consequently result in reduced antibiofilm activity of [Cu(DDC)_2_-liposomes + Cu^2+^-liposomes], the penetration of fluorescently-labelled liposomes into the biofilm should be determined using microscopical analysis [61,62] and the antibiofilm activity of non-PEGylated [Cu(DDC)_2_-liposomes + Cu^2+^-liposomes] should be investigated. As hydrophilic PEG polymers integration on the surface of Cu(DDC)_2_-liposomes is necessary for superior drug to lipid ratio and improvement of colloidal stability during storage compared to non-PEGylated Cu(DDC)_2_-liposomes [40] and [Cu(DDC)_2_-liposomes + free Cu^2+^] showed high antibiofilm activity against MRSA and *S. epidermidis*, incorporating Cu(DDC)_2_ into PEGylated liposomes is a water-soluble alternative for a potential application on surgical site infections.

### 3.5 *In vivo* toxicity and antimicrobial activity of liposomal DDC^−^ + Cu^2+^

*G. mellonella* is an invertebrate infection model that is cost- and time-efficient, can mimic physiological conditions of mammals, such as temperature of 37 °C, and expresses a cellular and humoral innate immune system [63]. This immune system is capable of recognizing pathogens and recruiting hemocytes to engulf pathogens and produce reactive oxygen species and antimicrobial peptides [[64], [65], [66]]. This model is in use for investigating pathogen virulence, for determining pharmacokinetic properties of antimicrobial agents and *in vivo* screening for antimicrobial activity and toxicity [[66], [67], [68], [69]]. Efficacy and toxicity of antibiotics in *G. mellonella* infection models were reported to empirically support the observed effects of antibiotics in murine infection models and antibiotic susceptibility in humans [70].

**Fig. 5:**
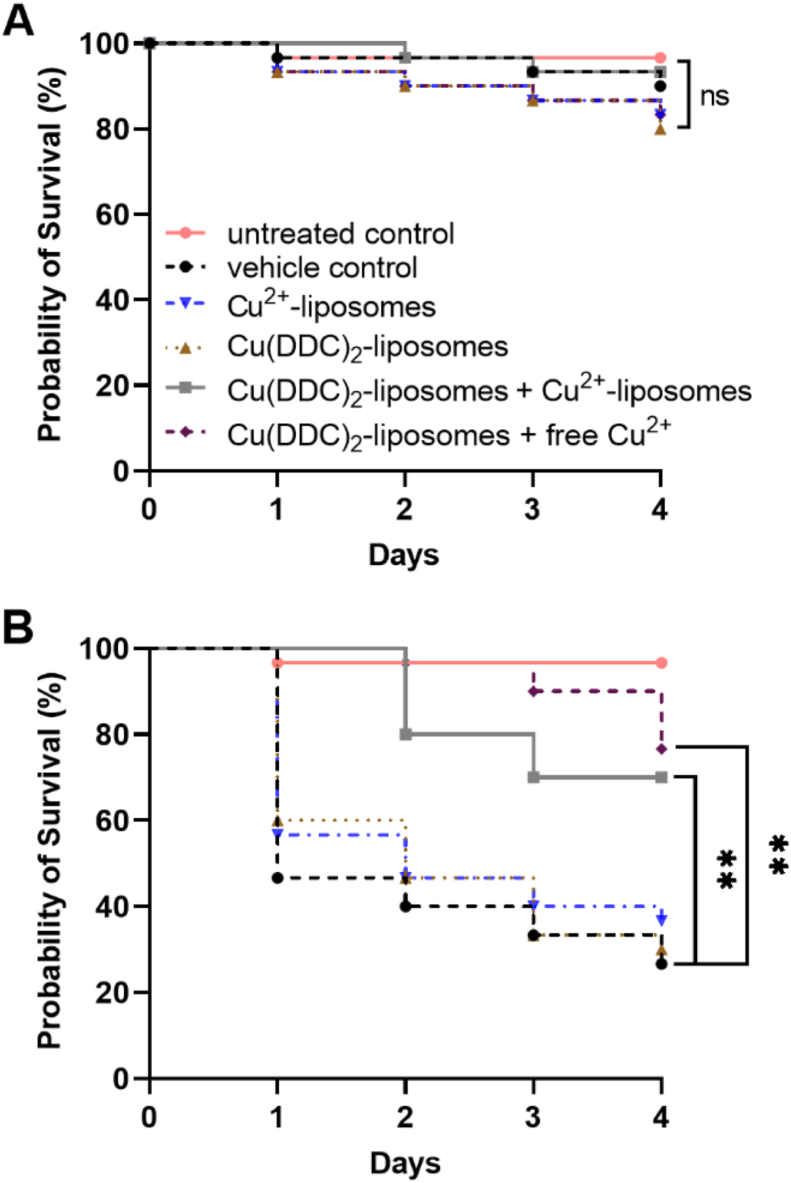
Effect of Cu^2+^-liposomes (blue), Cu(DDC)_2_-liposomes (brown), [Cu(DDC)_2_-liposomes + Cu^2+^-liposomes] (grey) and [Cu(DDC)_2_-liposomes + free Cu^2+^] (purple) on (A) the probability of *Galleria mellonella* survival (30/group; n = 180; ns = p > 0.05) and on (B) probability of survival of *Galleria mellonella* infected with *S. epidermidis* ATCC 35984 (30/group; n = 180; **p < 0.01). Vehicle = 0.9% saline (black); control = untreated, uninfected (pink). The concentrations of Cu(DDC)_2_-liposomes and Cu^2+^-liposomes correspond to 350 μM diethyldithiocarbamate (DDC^−^) and 1280 μM Cu^2+^, respectively. The combination of [Cu(DDC)_2_-liposomes + Cu^2+^-liposomes] and [Cu(DDC)_2_-liposomes + free Cu^2+^] represent a ratio of [1:6.2 mol] and correspond to 350 μM DDC^−^ + 1280 μM Cu^2+^. (For interpretation of the references to color in this figure legend, the reader is referred to the Web version of this article.)

To investigate potential toxic effects of the liposomes *in vivo, G. mellonella* larvae were exposed to liposomes and the survival was monitored over 4 days. Injection with Cu(DDC)_2_-liposomes, Cu^2+^-liposomes, the combination of [Cu(DDC)_2_-liposomes + Cu^2+^-liposomes] and the combination of [Cu(DDC)_2_-liposomes + free Cu^2+^] showed similar survival rates as the vehicle control (0.9% saline) and the untreated larvae, indicating no treatment toxicity in *G. mellonella* (Fig. 5A). Likewise, injection of free Cu^2+^ (concentration within larvae 128 μM) has been previously shown to be not toxic to *G. mellonella* larvae [31].

To assess the antimicrobial activity of [Cu(DDC)_2_-liposomes + Cu^2+^-liposomes] and [Cu(DDC)_2_-liposomes + free Cu^2+^] *in vivo*, the survival of *S. epidermidis*-infected *G. mellonella* was determined over 4 days (Fig. 5B). In *S. epidermidis-*infected larvae, treatment with Cu(DDC)_2_-liposomes or Cu^2+^-liposomes resulted in a low survival rate, similar to the vehicle control (*p* > 0.05). However, *S. epidermidis*-infected and [Cu(DDC)_2_-liposomes + Cu^2+^-liposomes] or [Cu(DDC)_2_-liposomes + free Cu^2+^] treated larvae showed a significantly higher survival rate compared to *S. epidermidis*-infected, saline treated larvae (*p* = 0.0018 and *p* = 0.0015, respectively). Moreover, the survival rates of both *S. epidermidis*-infected larvae treated with either [Cu(DDC)_2_-liposomes + Cu^2+^-liposomes] or [Cu(DDC)_2_-liposomes + free Cu^2+^] were significantly higher compared to treatment with Cu(DDC)_2_-liposomes alone (*p* = 0.0048 and *p* = 0.0015, respectively) or Cu^2+^-liposomes alone (*p* = 0.0203 and *p* = 0.0015, respectively). Notably, the substantial increase in survival of the *S. epidermidis*-infected, [Cu(DDC)_2_-liposomes + free Cu^2+^] treated larvae showed no significant difference to the survival rate of uninfected, untreated larvae (*p* > 0.05). While treatment with free Cu^2+^ previously showed no effect on *S. epidermidis*-infected larvae [31], treatment with [Cu(DDC)_2_-liposomes + free Cu^2+^] indicated efficacy against *S. epidermidis in vivo*.

Interestingly, the [Cu(DDC)_2_-liposomes + Cu^2+^-liposomes] combination significantly increased the survival rate of *S. epidermidis*-infected *G. mellonella* larvae, despite showing no antibiofilm activity *in vitro*. This increase in *S. epidermidis*-infected larvae survival was not significantly different to the [Cu(DDC)_2_-liposomes + free Cu^2+^] combination (*p* > 0.05). Consequently, the Cu^2+^-liposomes released their content *in vivo*, rendered excess Cu^2+^ available and resulted in antibacterial activity. However, *G. mellonella* larvae were injected with bacteria and liposomes simultaneously, not allowing for *in vivo* formation of biofilms before treatment. Therefore, the *in vivo* activity of [Cu(DDC)_2_-liposomes + Cu^2+^-liposomes] might be limited to planktonic bacteria. In addition, survival of *S. epidermidis*-infected larvae, treated with Cu(DDC)_2_-liposomes alone was not significantly different to the survival rate of *S. epidermidis*-infected, untreated larvae, validating previously determined effects of free Cu(DDC)_2_ + Cu^2+^ in *S. epidermidis*-infected larvae, where excess of Cu^2+^ was crucial for antibacterial activity. Moreover, absence of toxicity of Cu(DDC)_2_-liposomes and Cu^2+^-liposomes in *G. mellonella* larvae are in line with previous toxicity results of free Cu(DDC)_2_ + Cu^2+^ in *G. mellonella* and cell culture studies [31]. Consequently, the lack of toxicity and high efficacy of liposomal Cu(DDC)_2_ + Cu^2+^ observed in the *G. mellonella* model justify progressing to a mammalian *in vivo* infection model for pharmacological testing.

## 4 Discussion

We previously reported antibacterial and cytotoxic results of Cu(DDC)_2_ + Cu^2+^ against *S. aureus* and *S. epidermidis in vitro* and in *G. mellonella* larvae [31]. While the antibiofilm activity of Cu(DDC)_2_ + Cu^2+^ was determined in an *in vitro* biofilm model that is sufficient for an initial high throughput screening of novel antimicrobial drugs [31], this model is limited by the lack of resemblance to the microenvironment present in a human wound. Specific factors, such as wound exudate, host tissue, access to nutrients, formation of a biofilm gradient, presence of multiple bacterial species, inflammatory responses, and the immune system, influence the progression of a biofilm infection and the wound healing process [32]. By investigating the efficacy of antimicrobial compounds in physiologically relevant *in vitro* biofilm models of surgical site infections, instabilities of the drug or interactions with wound components can be detected and addressed to increase animal study validity before progressing to costly animal studies [48]. Although Cu(DDC)_2_ + Cu^2+^ preserved significant antibiofilm activity in challenging host-mimicking conditions, many factors present in an infected surgical wound, such as multiple bacterial species, the inflammatory response and the immune system were not incorporated in these *in vitro* models and can alter the outcome of future *in vivo* studies. Here, the effects of Cu(DDC)_2_ + Cu^2+^ on biofilms of the artificial dermis assay were diminished by the low water solubility of Cu(DDC)_2_ and by possible interactions with matrix components, which significantly limits the clinical application of the free compounds and shows the importance of an appropriate drug delivery system. By narrowing the gap between *in vitro* results and *in vivo* translation, we comply with the 3Rs principles by Russell et al. [71] to improve the welfare of animals used for research.

While Cu(DDC)_2_ showed *in vitro* activity against Mycobacteria [25], Streptococci [30,72], and Mycoplasma [73], the antibacterial effects have yet to be confirmed in animal models. In contrast, the research on Cu(DDC)_2_ as cancer treatment has progressed to *in vivo* experiments and first clinical trials. The application of Cu(DDC)_2_ in clinical trials is based on the separate oral administration of disulfiram and copper ions and the *in-situ* formation of Cu(DDC)_2_ [74]. However, poor biostability and solubility of disulfiram and Cu(DDC)_2_ often limit the treatment efficacy [51]. Alternative strategies are based on the encapsulation of Cu(DDC)_2_ into nanocarrier, such as micelles [75], cyclodextrins [76] and liposomes [39,77,78]. Here, Cu^2+^-liposomes and Cu(DDC)_2_-liposomes composed of DSPC, cholesterol and DSPE-mPEG_2000_ were investigated, as characteristics, including size, PDI, imaging, drug-to-lipid ratio and stability were described by Hartwig et al. [40] and Wehbe et al. [39] and freeze-drying of the liposomes enabled prolonged storage [79]. In addition, intravenous administration of 12.5 mg/kg modified PEGylated Cu(DDC)_2_-liposomes (without cholesterol) and 8 mg/kg of Cu(DDC)_2_-liposomes composed of DSPC and cholesterol were well tolerated in mice [39]. However, Wehbe et al. [39] only investigated the safety of Cu(DDC)_2_-liposomes and not the combination of [Cu(DDC)_2_-liposomes + Cu^2+^-liposomes] or [Cu(DDC)_2_-liposomes + free Cu^2+^], which is necessary for the antibiofilm activity. Furthermore, the outcome of *in vivo* safety experiments could be altered by the different lipid composition of the PEGylated liposomes and the non-PEGylated liposomes, due to changes in circulation lifetime after intravenous administration [39]. While the non-PEGylated liposomes were not investigated because of instabilities during storage [40], the PEGylated Cu(DDC)_2_-liposomes with cholesterol were stable and showed no toxicity in *G. mellonella* at 6.4 mg/kg. *G. mellonella* larvae are a good indicator for toxicity and efficacy before progressing to mammalian studies, but the mechanisms of toxicity of the tested compounds can be altered by lack of mammal-specific metabolization processes. Therefore, the combined results of *G. mellonella* and cell assay studies are a predictor of low toxicity of antimicrobial agents but do not replace safety experiments in mammals [70,80].

## 5 Conclusion

The Cu(DDC)_2_ + Cu^2+^ combination at concentrations of 35 μM DDC^−^ + 128 μM Cu^2+^ reduced the bacterial load of MRSA and *S. epidermidis* biofilms in an implant and wound model *in vitro*. In addition, the low water solubility of Cu(DDC)_2_ was overcome by incorporating the agents into liposomal carriers. Liposomal Cu(DDC)_2_ + Cu^2+^ showed antibiofilm activity *in vitro* against MRSA and *S. epidermidis* and *in vivo* efficacy against *S. epidermidis*, while being non-toxic. Therefore, the Cu(DDC)_2_ + Cu^2+^ combination represents a promising treatment strategy against *S. aureus* and *S. epidermidis* biofilm infections. Future studies will investigate the safety and efficacy of liposomal Cu(DDC)_2_ + Cu^2+^ in a mammalian model of wound infection.

## CRediT authorship contribution statement

**Laurine Kaul:** Conceptualization, Formal analysis, Investigation, Writing – original draft, preparation. **Adrian I. Abdo:** Formal analysis, Writing – review & editing. **Tom Coenye:** Methodology, Writing – review & editing. **Simon Swift:** Methodology, Writing – review & editing. **Andrew Zannettino:** Writing – review & editing, Supervision. **Regine Süss:** Conceptualization, Resources, Writing – review & editing, Supervision. **Katharina Richter:** Conceptualization, Investigation, Resources, Writing – review & editing, Supervision.

## Declaration of competing interest

The authors declare the following financial interests/personal relationships which may be considered as potential competing interests: Katharina Richter reports financial support was provided by 10.13039/501100000925National Health and Medical Research Council. Katharina Richter reports financial support was provided by 10.13039/100009727The Hospital Research Foundation. Laurine Kaul reports financial support was provided by 10.13039/501100001159Australian Society for Microbiology. Katharina Richter has patent #PCT/AU2020/050,661 issued to University of Adelaide. Tom Coenye is on the editorial board of the journal Biofilm - TC.

## Data Availability

No data was used for the research described in the article.

## References

[1] Andersen B.M., Andersen B.M. (2019). Prevention and control of infections in hospitals.

[2] World Health Organization (2016).

[3] Andersson R., Søreide K., Ansari D. (2021). Surgical infections and antibiotic stewardship: in need for new directions. Scand J Surg.

[4] Wilson R.B., Farooque Y. (2022). Risks and prevention of surgical site infection after hernia mesh repair and the predictive utility of ACS-NSQIP. J Gastrointest Surg.

[5] Costa A.C.D., Santa-Cruz F., Ferraz Á A.B. (2021). What's new in infection on surgical site and antibioticoprophylaxis in surgery?. Arq Bras Cir Dig.

[6] Mangram A.J., Horan T.C., Pearson M.L., Silver L.C., Jarvis W.R. (1999). Hospital infection control practices advisory committee. Guideline for prevention of surgical site infection, 1999. Infect Control Hosp Epidemiol.

[7] Owens C.D., Stoessel K. (2008). Surgical site infections: epidemiology, microbiology and prevention. J Hosp Infect.

[8] Mellinghoff S.C., Vehreschild J.J., Liss B.J., Cornely O.A. (2018). Epidemiology of surgical site infections with Staphylococcus aureus in europe: protocol for a retrospective, multicenter study. JMIR Res Protoc.

[9] Iskandar K., Sartelli M., Tabbal M., Ansaloni L., Baiocchi G.L., Catena F. (2019). Highlighting the gaps in quantifying the economic burden of surgical site infections associated with antimicrobial-resistant bacteria. World J Emerg Surg.

[10] Percival S.L. (2017). Importance of biofilm formation in surgical infection. Br J Surg.

[11] Hoffmann J.P., Friedman J.K., Wang Y., McLachlan J.B., Sammarco M.C., Morici L.A. (2020). In situ treatment with novel microbiocide inhibits methicillin resistant Staphylococcus aureus in a murine wound infection model. Front Microbiol.

[12] Humphreys H., Becker K., Dohmen P.M., Petrosillo N., Spencer M., van Rijen M. (2016). Staphylococcus aureus and surgical site infections: benefits of screening and decolonization before surgery. J Hosp Infect.

[13] Costerton J.W., Stewart P.S., Greenberg E.P. (1999). Bacterial biofilms: a common cause of persistent infections. Science.

[14] Mah T.F., O'Toole G.A. (2001). Mechanisms of biofilm resistance to antimicrobial agents. Trends Microbiol.

[15] Anderson D.J., Sexton D.J., Kanafani Z.A., Auten G., Kaye K.S. (2007). Severe surgical site infection in community hospitals: epidemiology, key procedures, and the changing prevalence of methicillin-resistant Staphylococcus aureus. Infect Control Hosp Epidemiol.

[16] Ellis P.M., Dronsfield A.T. (2013). Antabuse's diamond anniversary: still sparkling on?. Drug Alcohol Rev.

[17] Assolini J.P., Tomiotto-Pellissier F., da Silva Bortoleti B.T., Gonçalves M.D., Sahd C.S., Carloto A.C.M. (2020). Diethyldithiocarbamate encapsulation reduces toxicity and promotes leishmanicidal effect through apoptosis-like mechanism in promastigote and ROS production by macrophage. J Drug Target.

[18] Almeida-Silva J., Menezes D.S., Fernandes J.M.P., Almeida M.C., Vasco-Dos-Santos D.R., Saraiva R.M. (2022). The repositioned drugs disulfiram/diethyldithiocarbamate combined to benznidazole: searching for Chagas disease selective therapy, preventing toxicity and drug resistance. Front Cell Infect Microbiol.

[19] Rennar G.A., Gallinger T.L., Mäder P., Lange-Grünweller K., Haeberlein S., Grünweller A. (2022). Disulfiram and dithiocarbamate analogues demonstrate promising antischistosomal effects. Eur J Med Chem.

[20] Xu L., Tong J., Wu Y., Zhao S., Lin B.L. (2021). A computational evaluation of targeted oxidation strategy (TOS) for potential inhibition of SARS-CoV-2 by disulfiram and analogues. Biophys Chem.

[21] Shanholtzer C.N., Rice C., Watson K., Carreon H., Long T.E. (2022). Effect of copper on the antifungal activity of disulfiram (Antabuse®) in fluconazole-resistant Candida strains. Med Mycol.

[22] Harrison J.J., Turner R.J., Ceri H. (2007). A subpopulation of Candida albicans and Candida tropicalis biofilm cells are highly tolerant to chelating agents. FEMS Microbiol Lett.

[23] De Brucker K., Bink A., Meert E., Cammue B.P., Thevissen K. (2013). Potentiation of antibiofilm activity of amphotericin B by superoxide dismutase inhibition. Oxid Med Cell Longev.

[24] Byrne S.T., Gu P., Zhou J., Denkin S.M., Chong C., Sullivan D. (2007). Pyrrolidine dithiocarbamate and diethyldithiocarbamate are active against growing and nongrowing persister Mycobacterium tuberculosis. Antimicrob Agents Chemother.

[25] Dalecki A.G., Haeili M., Shah S., Speer A., Niederweis M., Kutsch O. (2015). Disulfiram and copper ions kill Mycobacterium tuberculosis in a synergistic manner. Antimicrob Agents Chemother.

[26] Nishimori I., Vullo D., Minakuchi T., Scozzafava A., Osman S.M., AlOthman Z. (2014). Anion inhibition studies of two new β-carbonic anhydrases from the bacterial pathogen Legionella pneumophila. Bioorg Med Chem Lett.

[27] Kaul L., Süss R., Zannettino A., Richter K. (2021). The revival of dithiocarbamates: from pesticides to innovative medical treatments. iScience.

[28] Cvek B., Milacic V., Taraba J., Dou Q.P. (2008). Ni(II), Cu(II), and Zn(II) diethyldithiocarbamate complexes show various activities against the proteasome in breast cancer cells. J Med Chem.

[29] Tawari P.E., Wang Z., Najlah M., Tsang C.W., Kannappan V., Liu P. (2015). The cytotoxic mechanisms of disulfiram and copper(ii) in cancer cells. Toxicol Res.

[30] Menghani S.V., Rivera A., Neubert M., Hagerty J.R., Lewis L., Galgiani J.N. (2021). Demonstration of N,N-dimethyldithiocarbamate as a copper-dependent antibiotic against multiple upper respiratory tract pathogens. Microbiol Spectr.

[31] Kaul L., Abdo A.I., Coenye T., Krom B.P., Hoogenkamp M.A., Zannettino A.C.W. (2022). The combination of diethyldithiocarbamate and copper ions is active against Staphylococcus aureus and Staphylococcus epidermidis biofilms in vitro and in vivo. Front Microbiol.

[32] Thaarup I.C., Bjarnsholt T. (2020). Current in vitro biofilm-infected chronic wound models for developing new treatment possibilities. Adv Wound Care.

[33] Wehbe M., Anantha M., Backstrom I., Leung A., Chen K., Malhotra A. (2016). Nanoscale reaction vessels designed for synthesis of copper-drug complexes suitable for preclinical development. PLoS One.

[34] Han J., Liu L., Yue X., Chang J., Shi W., Hua Y. (2013). A binuclear complex constituted by diethyldithiocarbamate and copper(I) functions as a proteasome activity inhibitor in pancreatic cancer cultures and xenografts. Toxicol Appl Pharmacol.

[35] Allensworth J.L., Evans M.K., Bertucci F., Aldrich A.J., Festa R.A., Finetti P. (2015). Disulfiram (DSF) acts as a copper ionophore to induce copper-dependent oxidative stress and mediate anti-tumor efficacy in inflammatory breast cancer. Mol Oncol.

[36] Chen W., Yang W., Chen P., Huang Y., Li F. (2018). Disulfiram copper nanoparticles prepared with a stabilized metal ion ligand complex method for treating drug-resistant prostate cancers. ACS Appl Mater Interfaces.

[37] Meng Z., Wang H., Fang X., Liu Z., Yang Z., Yong J. (2021). Surface decoration via physical interaction of cupric diethyldithiocarbamate nanocrystals and its impact on biodistribution and tumor targeting. ACS Appl Mater Interfaces.

[38] Ren L., Feng W., Shao J., Ma J., Xu M., Zhu B.Z. (2020). Diethyldithiocarbamate-copper nanocomplex reinforces disulfiram chemotherapeutic efficacy through light-triggered nuclear targeting. Theranostics.

[39] Wehbe M., Anantha M., Shi M., Leung A.W., Dragowska W.H., Sanche L. (2017). Development and optimization of an injectable formulation of copper diethyldithiocarbamate, an active anticancer agent. Int J Nanomed.

[40] Hartwig F., Köll-Weber M., Süss R. (2021). Preclinical in vitro studies with 3D spheroids to evaluate Cu(DDC)2 containing liposomes for the treatment of neuroblastoma. Pharmaceutics.

[41] Brackman G., Garcia-Fernandez M.J., Lenoir J., De Meyer L., Remon J.-P., De Beer T. (2016). Dressings loaded with cyclodextrin–hamamelitannin complexes increase Staphylococcus aureus susceptibility toward antibiotics both in single as well as in mixed biofilm communities. Macromol Biosci.

[42] Richter K., Thomas N., Claeys J., McGuane J., Prestidge C.A., Coenye T. (2017). A topical hydrogel with deferiprone and gallium-protoporphyrin targets bacterial iron metabolism and has antibiofilm activity. Antimicrob Agents Chemother.

[43] Richter K., Ramezanpour M., Thomas N., Prestidge C.A., Wormald P.J., Vreugde S. (2016). Mind "De GaPP": in vitro efficacy of deferiprone and gallium-protoporphyrin against Staphylococcus aureus biofilms. Int Forum Allergy Rhinol.

[44] Van den Driessche F., Rigole P., Brackman G., Coenye T. (2014). Optimization of resazurin-based viability staining for quantification of microbial biofilms. J Microbiol Methods.

[45] Patiniott P., Jacombs A., Kaul L., Hu H., Warner M., Klosterhalfen B. (2022). Are late hernia mesh complications linked to staphylococci biofilms?. Hernia.

[46] Jacombs A.S.W., Karatassas A., Klosterhalfen B., Richter K., Patiniott P., Hensman C. (2020). Biofilms and effective porosity of hernia mesh: are they silent assassins?. Hernia.

[47] Engelsman A.F., van der Mei H.C., Busscher H.J., Ploeg R.J. (2008). Morphological aspects of surgical meshes as a risk factor for bacterial colonization. Br J Surg.

[48] Grassi L., Batoni G., Ostyn L., Rigole P., Van den Bossche S., Rinaldi A.C. (2019). The antimicrobial peptide lin-SB056-1 and its dendrimeric derivative prevent pseudomonas aeruginosa biofilm formation in physiologically relevant models of chronic infections. Front Microbiol.

[49] Wehbe M., Chernov L., Chen K., Bally M.B. (2016). PRCosomes: pretty reactive complexes formed in liposomes. J Drug Target.

[50] Sajithlal G.B., Chithra P., Chandrakasan G. (1999). An in vitro study on the role of metal catalyzed oxidation in glycation and crosslinking of collagen. Mol Cell Biochem.

[51] Johansson B. (1992). A review of the pharmacokinetics and pharmacodynamics of disulfiram and its metabolites. Acta Psychiatr Scand.

[52] Hassan G., Forsman N., Wan X., Keurulainen L., Bimbo L.M., Stehl S. (2020). Non-leaching, highly biocompatible nanocellulose surfaces that efficiently resist fouling by bacteria in an artificial dermis model. ACS Appl Bio Mater.

[53] Grassi L., Pompilio A., Kaya E., Rinaldi A.C., Sanjust E., Maisetta G. (2020). The anti-microbial peptide (lin-SB056-1)2-K reduces pro-inflammatory cytokine release through interaction with Pseudomonas aeruginosa lipopolysaccharide. Antibiotics.

[54] Caputo F., Clogston J., Calzolai L., Rösslein M., Prina-Mello A. (2019). Measuring particle size distribution of nanoparticle enabled medicinal products, the joint view of EUNCL and NCI-NCL. A step by step approach combining orthogonal measurements with increasing complexity. J Contr Release.

[55] Danaei M., Dehghankhold M., Ataei S., Hasanzadeh Davarani F., Javanmard R., Dokhani A. (2018). Impact of particle size and polydispersity index on the clinical applications of lipidic nanocarrier systems. Pharmaceutics.

[56] Wang D.-Y., van der Mei H.C., Ren Y., Busscher H.J., Shi L. (2020). Lipid-based antimicrobial delivery-systems for the treatment of bacterial infections. Front Chem.

[57] Forier K., Raemdonck K., De Smedt S.C., Demeester J., Coenye T., Braeckmans K. (2014). Lipid and polymer nanoparticles for drug delivery to bacterial biofilms. J Contr Release.

[58] Rukavina Z., Ž Vanić (2016). Current trends in development of liposomes for targeting bacterial biofilms. Pharmaceutics.

[59] Ibaraki H., Kanazawa T., Chien W.-Y., Nakaminami H., Aoki M., Ozawa K. (2020). The effects of surface properties of liposomes on their activity against Pseudomonas aeruginosa PAO-1 biofilm. J Drug Deliv Sci Technol.

[60] Verhoef J.J., Anchordoquy T.J. (2013). Questioning the use of PEGylation for drug delivery. Drug Deliv Transl Res.

[61] Ahmed K., Gribbon P.N., Jones M.N. (2002). The application of confocal microscopy to the study of liposome adsorption onto bacterial biofilms. J Liposome Res.

[62] Dong D., Thomas N., Thierry B., Vreugde S., Prestidge C.A., Wormald P.-J. (2015). Distribution and inhibition of liposomes on Staphylococcus aureus and Pseudomonas aeruginosa biofilm. PLoS One.

[63] Maurer E., Hörtnagl C., Lackner M., Grässle D., Naschberger V., Moser P. (2019). Galleria mellonella as a model system to study virulence potential of mucormycetes and evaluation of antifungal treatment. Med Mycol.

[64] Senior N.J., Titball R.W. (2020). Isolation and primary culture of Galleria mellonella hemocytes for infection studies. F1000Res.

[65] Bergin D., Reeves E.P., Renwick J., Wientjes F.B., Kavanagh K. (2005). Superoxide production in *Galleria mellonella* hemocytes: identification of proteins homologous to the NADPH oxidase complex of human neutrophils. Infect Immun.

[66] Tsai C.J.-Y., Loh J.M.S., Proft T. (2016). Galleria mellonella infection models for the study of bacterial diseases and for antimicrobial drug testing. Virulence.

[67] Sheehan G., Dixon A., Kavanagh K. (2019). Utilization of Galleria mellonella larvae to characterize the development of Staphylococcus aureus infection. Microbiology (Read).

[68] Brackman G., Cos P., Maes L., Nelis H.J., Coenye T. (2011). Quorum sensing inhibitors increase the susceptibility of bacterial biofilms to antibiotics in vitro and in vivo. Antimicrob Agents Chemother.

[69] Fuchs B.B., O'Brien E., Khoury J.B., Mylonakis E. (2010). Methods for using Galleria mellonella as a model host to study fungal pathogenesis. Virulence.

[70] Ignasiak K., Maxwell A. (2017). Galleria mellonella (greater wax moth) larvae as a model for antibiotic susceptibility testing and acute toxicity trials. BMC Res Notes.

[71] Russell W.M.S., Burch R.L. (1959).

[72] Saputo S., Faustoferri R.C., Quivey R.G. (2018). A drug repositioning approach reveals that Streptococcus mutans is susceptible to a diverse range of established antimicrobials and nonantibiotics. Antimicrob Agents Chemother.

[73] Totten A.H., Crawford C.L., Dalecki A.G., Xiao L., Wolschendorf F., Atkinson T.P. (2019). Differential susceptibility of mycoplasma and ureaplasma species to compound-enhanced copper toxicity. Front Microbiol.

[74] Kannappan V., Ali M., Small B., Rajendran G., Elzhenni S., Taj H. (2021). Recent advances in repurposing disulfiram and disulfiram derivatives as copper-dependent anticancer agents. Front Mol Biosci.

[75] Kang X., Wang J., Huang C.H., Wibowo F.S., Amin R., Chen P. (2023). Diethyldithiocarbamate copper nanoparticle overcomes resistance in cancer therapy without inhibiting P-glycoprotein. Nanomedicine.

[76] Said Suliman A., Khoder M., Tolaymat I., Webster M., Alany R.G., Wang W. (2021). Cyclodextrin diethyldithiocarbamate copper II inclusion complexes: a promising chemotherapeutic delivery system against chemoresistant triple negative breast cancer cell lines. Pharmaceutics.

[77] Paun R.A., Dumut D.C., Centorame A., Thuraisingam T., Hajduch M., Mistrik M. (2022). One-step synthesis of nanoliposomal copper diethyldithiocarbamate and its assessment for cancer therapy. Pharmaceutics.

[78] Zheng Z., Zhang J., Jiang J., He Y., Zhang W., Mo X. (2020). Remodeling tumor immune microenvironment (TIME) for glioma therapy using multi-targeting liposomal codelivery. J Immunother Cancer.

[79] Kaul L., Grundmann C.E., Köll-Weber M., Löffler H., Weiz A., Zannettino A.C.W. (2022). A thermosensitive, chitosan-based hydrogel as delivery system for antibacterial liposomes to surgical site infections. Pharmaceutics.

[80] Allegra E., Titball R.W., Carter J., Champion O.L. (2018). Galleria mellonella larvae allow the discrimination of toxic and non-toxic chemicals. Chemosphere.

