# Supplementary material for: In vitro and in vivo evaluation of diethyldithiocarbamate with copper ions and its liposomal formulation for the treatment of Staphylococcus aureus and Staphylococcus epidermidis biofilms
Source: Biofilm. 2023 May 17;5:100130. doi: 10.1016/j.bioflm.2023.100130 (PMC10238467; doi:10.1016/j.bioflm.2023.100130)
Supplement: Multimedia component 1 [file mmc1.docx]

# Supplementary file

**Supplementary Figure 1:** Percentage of green fluorescence in confocal microscopy images of S. aureus ATCC 6538 biofilms on polyester or polypropylene meshes treated with Cu(DDC)_2_ + Cu^2+^ (grey) compared to untreated meshes (white). Quantification of images as green and red fluorescence. n=3-4; mean ± SD; 2-way ANOVA: **p< 0.01 indicate significant differences between Cu(DDC)_2_ + Cu^2+^ and untreated control by Šidák’s multiple comparison test; ns p> 0.05 indicate no significant differences between the polyester and the polypropylene mesh.
